# Acidic pH is essential for maintaining mast cell secretory granule homeostasis

**DOI:** 10.1038/cddis.2017.206

**Published:** 2017-05-11

**Authors:** Gunnar Pejler, Jun Mei Hu Frisk, Daniel Sjöström, Aida Paivandy, Helena Öhrvik

**Affiliations:** 1 Uppsala University, Department of Medical Biochemistry and Microbiology, Uppsala, Sweden; 2 Swedish University of Agricultural Sciences, Department of Anatomy, Physiology and Biochemistry, Uppsala, Sweden

## Abstract

It has been recognized for a long time that the secretory granules of mast cells are acidic, but the functional importance of maintaining an acidic pH in the mast cell granules is not fully understood. Here we addressed this issue by examining the effects of raising the pH of the mast cell secretory granules. Mast cells were incubated with bafilomycin A1, an inhibitor of the vacuolar-type ATPase proton pump. Supporting a role of vacuolar-type ATPase in mast cell granule acidification, bafilomycin A1 treatment caused a robust increase in granule pH. This was accompanied by marked effects on mast cell granules, including swelling and acquisition of vacuole-like morphology. Moreover, bafilomycin A1 caused extensive, yet selective effects on the granule content. These included aberrant processing of pro-carboxypeptidase A3 and a reduction in the level of intracellular histamine, the latter being accompanied by an increase in extracellular histamine. In contrast, the storage of *β*-hexosaminidase, a prototype lysosomal hydrolase known to be stored in mast cell granules, was not affected by abrogation of granule acidification. Moreover, bafilomycin A1 caused a reduction of tryptase enzymatic activity and appearance of tryptase degradation products. Tryptase inhibition prevented the formation of such degradation products, suggesting that the pH elevation causes tryptase to undergo autoproteolysis. Taken together, our findings reveal that mast cell secretory granule homeostasis is critically dependent on an acidic milieu.

Mast cells are hematopoietic cells with a large impact on various immune reactions including, in particular, allergic conditions but mast cells have also been implicated in numerous additional pathologies such as arthritis and cancer.^1^ A hallmark feature of mast cells is their large content of highly electron-dense secretory granules. These are filled with large amounts of different bioactive compounds such as various amines (e.g., histamine, serotonin), cytokines, proteoglycans of serglycin type and different mast cell-restricted proteases, the latter including chymase, tryptase and CPA3.^2, 3^ When mast cells undergo degranulation, typically induced by IgE receptor cross-linking^4^ or by engagement of the MRGPRX2/MRGPRB2 receptor,^5^ the contents of the granules are expelled to the exterior and can cause a profound inflammatory reaction.^2^

Mast cell granules share important features with lysosomes and can because of this be denoted as ‘secretory lysosomes’.^6^ For example, the mast cell granules contain typical lysosomal enzymes such as cysteine- and aspartic acid cathepsins, aryl sulfatase A, *β*-glucuronidase and *β*-hexosaminidase.^2^ Another similarity between lysosomes and secretory granules is that both of these organelles are acidic. In lysosomes, the acidic pH is important for ensuring that the lysosomal enzymes, which typically have an acidic pH optimum, are fully active to carry out degradation of compounds that have been delivered into this organelle. Moreover, the acidic pH optimum of the lysosomal enzymes ensures that their action is largely confined to this cellular compartment, whereas the neutral pH milieu of the extracellular space or the cytosol will cause their inactivation and thus confer protection from their potentially damaging effects.

Although it has been known for a long time that the mast cell granules have a low pH,^7, 8, 9^ the impact of the acidic pH milieu on mast cell granule functionality and homeostasis is not fully understood. In this study we addressed this issue. The strategy of the investigation was to impair the acidification of mast cell granules by inhibiting the proton-pumping mechanism, and as a tool for this purpose we used bafilomycin A1. Bafilomycin A1 is a specific inhibitor of vacuolar-type ATPase (V-ATPase)^10^ and considering that V-ATPase has a key role in the acidification of lysosomes,^11^ we reasoned that this enzyme could serve a corresponding function in regulating the pH of mast cell granules. Indeed, we show that bafilomycin A1 effectively raises the pH of mast cell secretory granules. Moreover, we demonstrate that the elevation of the pH in mast cell granules has profound effects on granule homeostasis, including marked morphological effects, as well as defects in the storage and activity of granule compounds. Hence, this investigation reveals a prominent role of acidic pH in regulating the homeostasis on mast cell secretory granules.

## Results

### V-ATPase is essential for mast cell granule acidification

**Figure 1:**
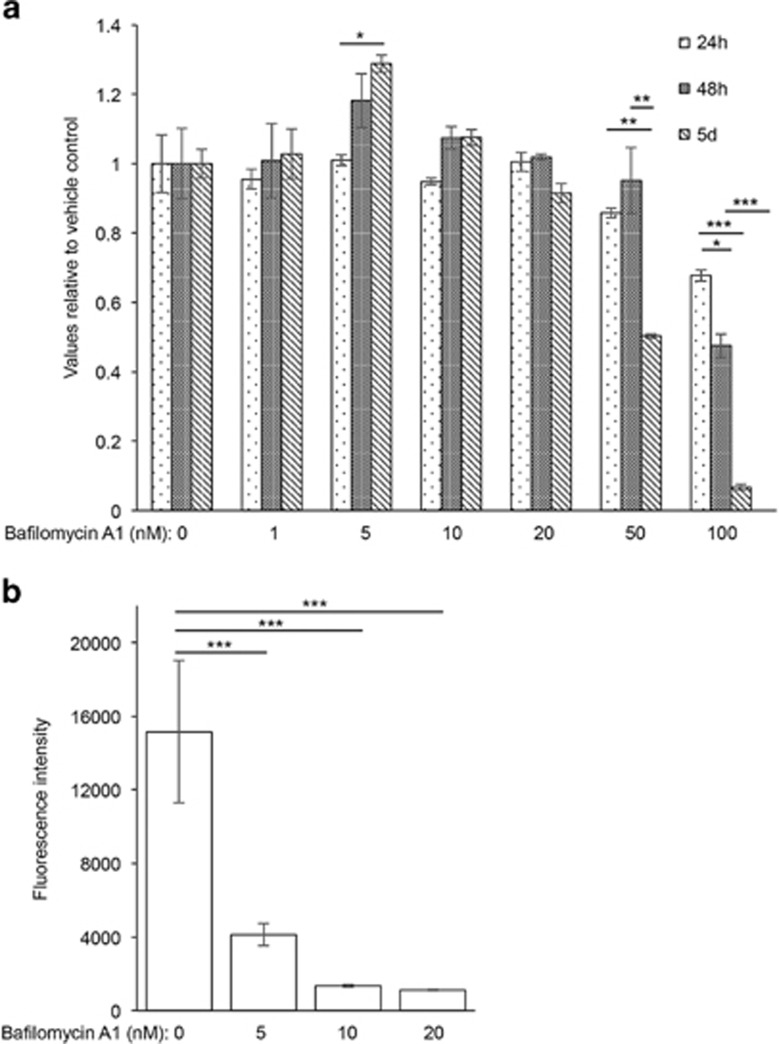
Effect of bafilomycin A1 on mast cell viability and granule acidification. (**a**) Mast cells (bone marrow-derived mast cells; BMMCs), 10 000 cells/well, were incubated in the absence or presence of bafilomycin A1 at the indicated concentrations and time periods, followed by assessment of viability using a fluorogenic viability assay test. Residual viability is given relative to vehicle-treated control cells. (**b**) BMMCs (0.44 × 10^6^ cells) were incubated for 3 h in the absence or presence of bafilomycin A1 at the indicated concentrations. Next, Lysosensor Blue DND-167 (1 *μ*M) was added to the cells followed by flow cytometry analysis. Results are representative of two individual experiments. Results are given as mean values±S.D. (*n*=3). *P<0.05, **P<0.01, ***P<0.001

For the purpose of assessing the role of acidic pH on the homeostasis of the mast cell secretory granules the strategy was to intervene with the granule acidification. For this purpose we used bafilomycin A1, an inhibitor of V-ATPase. As V-ATPase is known to have a prominent role in the acidification of lysosome-like organelles in numerous other cell types,^10, 11^ we reasoned that it was likely that this ATPase has an analogous function in the regulation of pH in mast cell secretory granules. To address this possibility, we assessed the effect of bafilomycin A1 on mast cells. At concentrations up to 50 nM, bafilomycin A1 was non-toxic to bone marrow-derived mast cells (BMMCs) at incubation times up to 48 h. However, after 5 days of incubation, bafilomycin A1 at 50 nM showed some toxicity. At higher concentrations (100 nM) profound toxicity was seen, in particular, after 5 days of culture (Figure 1a). Next, bafilomycin A1 was added to mast cells at non-toxic conditions (up to 20 nM) followed by the assessment of the pH of granules by using Lysosensor Blue, a dye that localizes to acidic intracellular compartments and produces strong fluorescence at low pH conditions. As shown in Figure 1b, addition of bafilomycin A1 at 20 nM (3h) to BMMCs caused a marked reduction in fluorescence, suggesting that the granule acidification is hampered. Hence, these findings suggest that V-ATPase is essential for granule acidification in mast cells.

### Impaired granule acidification disrupts mast cell granule morphology

**Figure 2:**
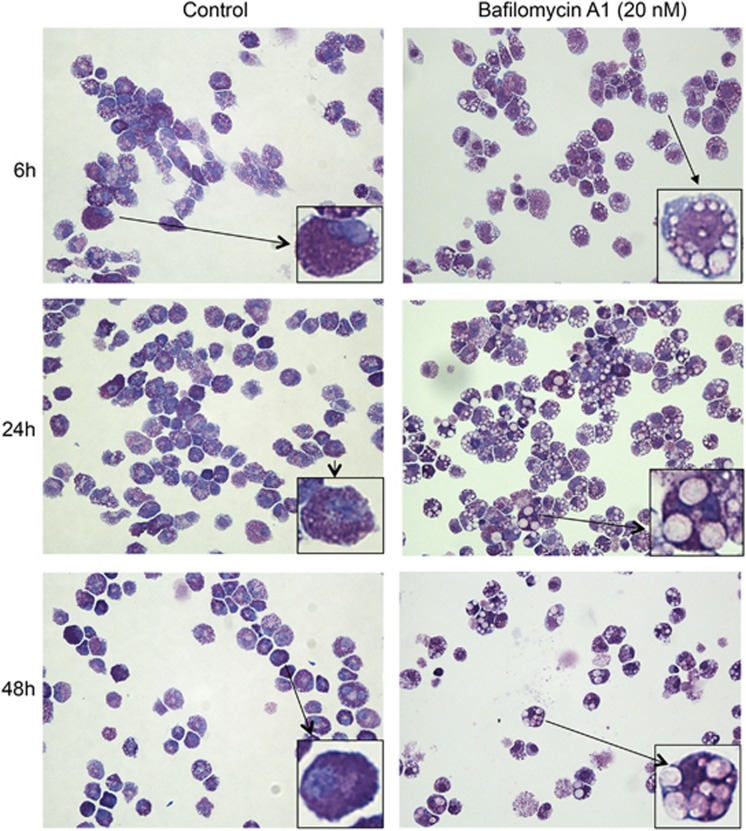
Impaired acidification causes aberrant granule morphology in mast cells. Mast cells (BMMCs; 0.5 × 10^6^ cells) were incubated in the absence or presence of 20 nM bafilomycin A1 at the indicated time periods, followed by preparation of cytospin slides (50 000 cells/slide) and May Grünwald/Giemsa staining. Images representative of three independent experiments are shown

To clarify the impact of acidic pH on mast cell granule homeostasis we first investigated the effect of hampered granule acidification on mast cell morphology. For this purpose we stained cytospin slides of either untreated or bafilomycin A1-treated BMMCs with May Grünwald/Giemsa (MGG), after incubation with bafilomycin A1 for up to 48 h. As depicted in Figure 2, bafilomycin A1 treatment (20 nM) caused marked effects on mast cell granule morphology already after 6 h of treatment. The most marked effect seen was a substantial swelling of granules, causing the granules to adopt a vacuole-like appearance. Typically, MGG-positive material tended to accumulate at the edges of the granules in bafilomycin A1-treated cells, whereas large regions of the granules appeared empty (MGG-negative). Upon prolonged treatment with bafilomycin A1, these effects were either sustained or even more pronounced (Figure 2). Similar, although somewhat less pronounced effects of bafilomycin A1 were seen also at lower concentrations (down to 5 nM; data not shown).

**Figure 3:**
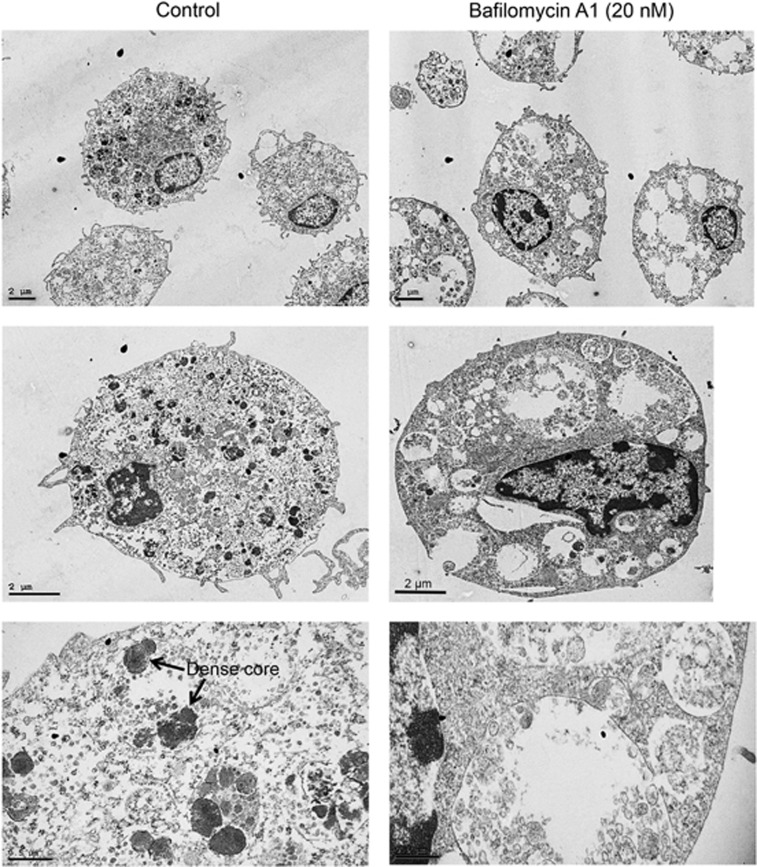
Transmission electron microscopy (TEM) analysis of the effects of bafilomycin A1 on mast cell morphology. Mast cells (4 × 10^6^ cells) were treated for 48 h with 20 nM bafilomycin A1 or vehicle, followed by TEM analysis. The upper panels show representative overviews of cells, whereas the lower panels represent enlarged images. Original magnifications: 5000 × (upper panels), 9000 × (middle panels), 40 000 × (lower panels)

To provide further insight into the effects of bafilomycin A1 on mast cell granules, transmission electron microscopy (TEM) analysis was conducted. In agreement with the dramatic morphological effects seen after MGG staining, TEM analysis revealed pronounced effects of impaired acidification on the mast cell granules (Figure 3). In non-treated (control) mast cells, granules typically contained electron-dense core structures interspersed with more electron translucent material (Figure 3). However, after bafilomycin A1 treatment, the content of electron-dense core structures within granules was profoundly reduced, accompanied by larger regions with electron translucent material. In agreement with the light microscopy analyses, TEM analysis showed that bafilomycin A1 treatment caused substantial swelling of the granules.

### *β*-hexosaminidase storage is independent of acidic granule pH

**Figure 4:**
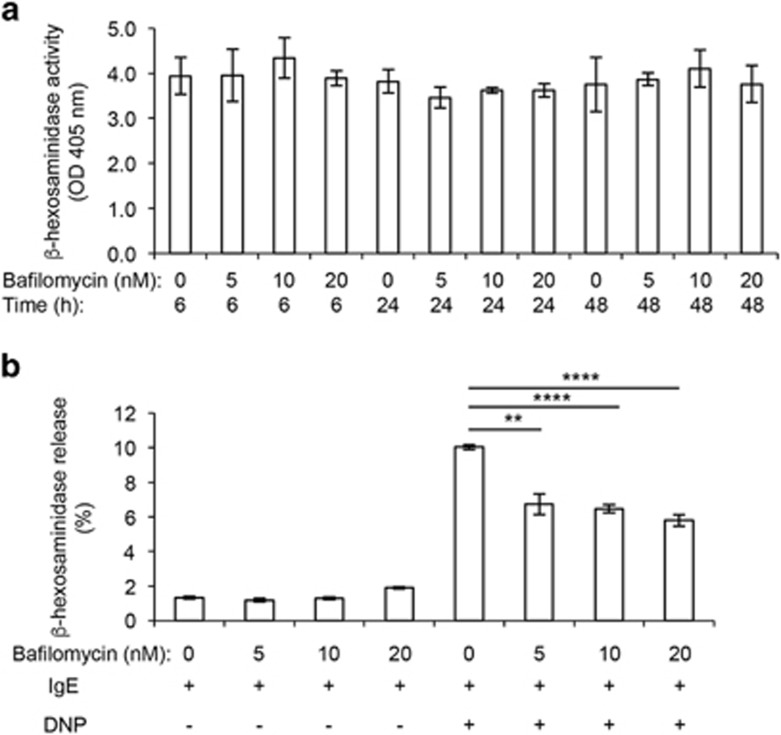
Effect of bafilomycin A1 on *β*-hexosaminidase storage and immunological release. (**a**) Mast cells (0.5 × 10^6^ cells) were incubated either in the absence or presence of bafilomycin A1 at the indicated concentrations and during the indicated time periods. Cells were pelleted and intracellular content of *β*-hexosaminidase activity was measured. (**b**) Mast cells (1 × 10^6^ cells/ml) were incubated for 24 h with the indicated concentrations of bafilomycin A1. Cells were then washed twice with PBS and further incubated in presence of either IgE alone (0.1 *μ*g/ml) or IgE (0.1 *μ*g/ml) + DNP (0.5 *μ*g/ml). After an additional incubation period (60 min), the amount of released *β*-hexosaminidase activity was measured in the cell culture supernatant to monitor the extent of mast cell degranulation. Results are representative of three individual experiments. Results are given as mean values±S.D. (*n*=3). **P<0.01, ****P<0.001

Considering the profound effects of impaired granule acidification on mast cell granule morphology, we next investigated the possibility that low pH has an impact on the storage of granule contents. To this end, we first assessed the effect of bafilomycin A1 treatment on *β*-hexosaminidase. *β*-hexosaminidase is a lysosomal enzyme that is present in the secretory granules of mast cells of all tissue locations and in all species, and the release of *β*-hexosaminidase is commonly used as a measure of the extent of mast cell degranulation in response to mast cell activating regimens. BMMCs were treated with bafilomycin A1 for various time periods followed by the assessment of residual intracellular *β*-hexosaminidase activity. However, it was evident that impaired acidification of the granules did not have any detectable impact on the amount of *β*-hexosaminidase stored (Figure 4a). Further experiments were conducted to assess whether prevention of granule acidification had an impact on mast cell functionality in terms of ability to degranulate. For this purpose, mast cells were immunologically activated through IgE receptor cross-linking followed by measurements of *β*-hexosaminidase release to the extracellular space. As seen in Figure 4b, bafilomycin A1-treated mast cells retained the ability to degranulate in response to IgE receptor cross-linking. However, the extent of *β*-hexosaminidase release was significantly reduced in comparison with vehicle-treated cells.

### Histamine storage in mast cell granules is dependent on acidic pH

**Figure 5:**
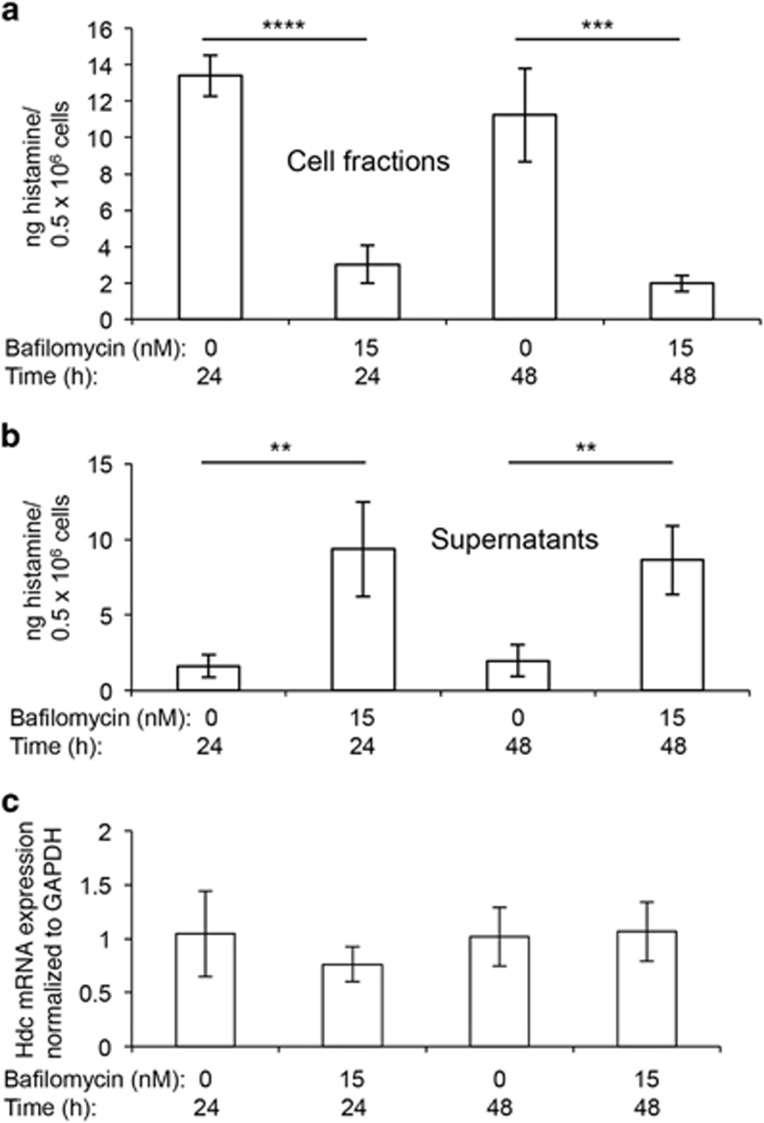
Histamine storage in mast cells is dependent on acidic granule pH. Mast cells (1 × 10^6^ cells/ml) were incubated for 24 or 48 h with 0, 5 or 15 nM bafilomycin A1. Histamine content in the cell pellets (**a**) and in the supernatants (**b**) was measured by ELISA. (**c**) Mast cells were incubated with bafilomycin A1 at the concentrations and time periods indicated. Cells were pelleted by centrifugation, followed by RNA isolation and qPCR analysis for content of mRNA coding for Hdc. The results are representative of two individual experiments. Results are given as mean values±S.D. (*n*=4). **P<0.01, ***P<0.001, ****P<0.001; ns, not significant

A hallmark feature of mast cells of all locations and species is their high content of histamine, a bioactive amine with profound effects on various parameters of inflammation. In contrast to its impact on *β*-hexosaminidase, bafilomycin A1 treatment caused a profound reduction in the ability of mast cells to store histamine intracellularly (Figure 5a), and this was accompanied by a large increase in the amount of histamine recovered from the cell culture medium (Figure 5b). Notably, the total histamine content (intracellular+extracellular) was approximately equal in vehicle-treated *versus* bafilomycin A1-treated cells, suggesting that the rate of histamine synthesis was not affected by bafilomycin A1. In agreement with this, the expression of the gene coding for histidine decarboxylase (Hdc), that is, the enzyme responsible for conversion of histidine to histamine, was not significantly affected by raising the granule pH (Figure 5c). Hence, a low pH of the granules is essential for the ability of mast cells to store histamine, and this is independent of effects on histamine biosynthesis.

### Aberrant processing of CPA3 in mast cell granules with impaired acidification

**Figure 6:**
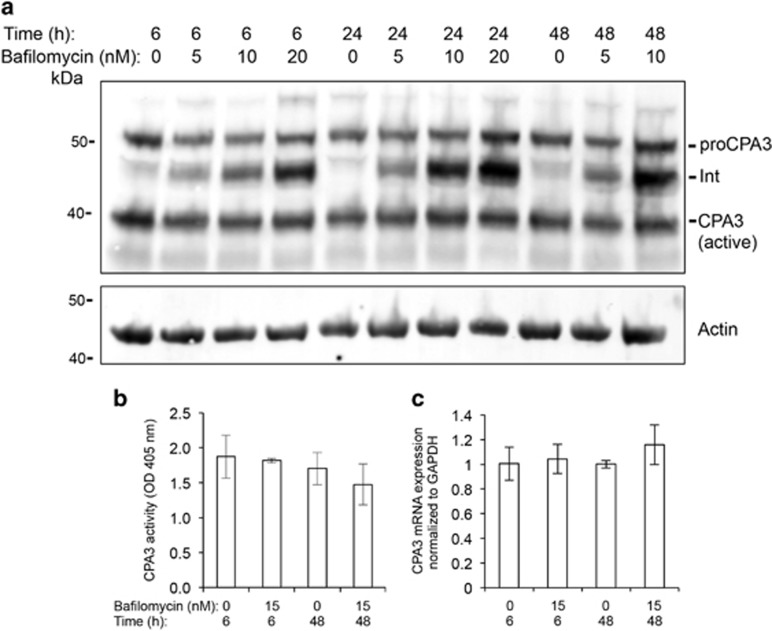
Aberrant processing of CPA3 in bafilomycin A1-treated mast cells. Mast cells (1 × 10^6^ cells/ml) were incubated with bafilomycin A1 at the concentrations and time periods indicated. (**a**) Cells were then recovered by centrifugation, followed by preparation of cell protein extracts and western blot analysis for CPA3 processing products using an anti-CPA3 antibody. The migration position of proCPA3 and the fully processed form (active) of CPA3 are indicated. Note the appearance of an intermediate processing form of CPA3 (Int) in cells treated with bafilomycin A1. *β*-actin was used as loading control. (**b**) Cell extracts from non-treated and bafilomycin A1-treated mast cells were analyzed for levels of CPA3 activity using a chromogenic substrate. Results are representative of five individual experiments. Results are given as mean values±S.D. (*n*=4). (**c**) RNA from non-treated and bafilomycin A1-treated mast cells was analyzed by qPCR for content of mRNA coding for the CPA3 gene (*Cpa3*). Results are given as mean±S.D. (*n*=3). The results are representative of three individual experiments

CPA3 is a major component of mast cell granules, and has an important role in various biological processes such as degradation of toxins.^12^ To clarify the role of acidic pH for CPA3 homeostasis, BMMCs were treated with bafilomycin A1 followed by the assessment of CPA3 protein by western blot analysis. This analysis revealed that the amount of fully processed CPA3 (active) was not affected by the impairment of granule acidification. Moreover, the levels of proCPA3 were approximately equal in non-treated *versus* bafilomycin A1-treated BMMCs (Figure 6a). However, bafilomycin A1 treatment caused a marked accumulation of a form of CPA3 with a molecular weight in between that of proCPA3 and fully processed CPA3, most likely corresponding to an intermediate product in the processing of proCPA3 (Figure 6a).

The data in Figure 6a suggest that the levels of fully processed CPA3 are not affected by pH elevation. However, this does not exclude that the pH elevation might affect the enzymatic activity of CPA3. To address this possibility, CPA3 enzymatic activity was measured in vehicle- and bafilomycin A1-treated mast cells. In line with the western blot analysis, bafilomycin A1 treatment did not cause any significant effect on the enzymatic activity of CPA3, although a tendency of decreased activity was seen in mast cells treated for 48 h with bafilomycin A1 (Figure 6b). Moreover, bafilomycin A1 did not affect the CPA3 gene expression (Figure 6c).

### Acidic pH is critical for maintaining tryptase activity and protein integrity in secretory granules

**Figure 7:**
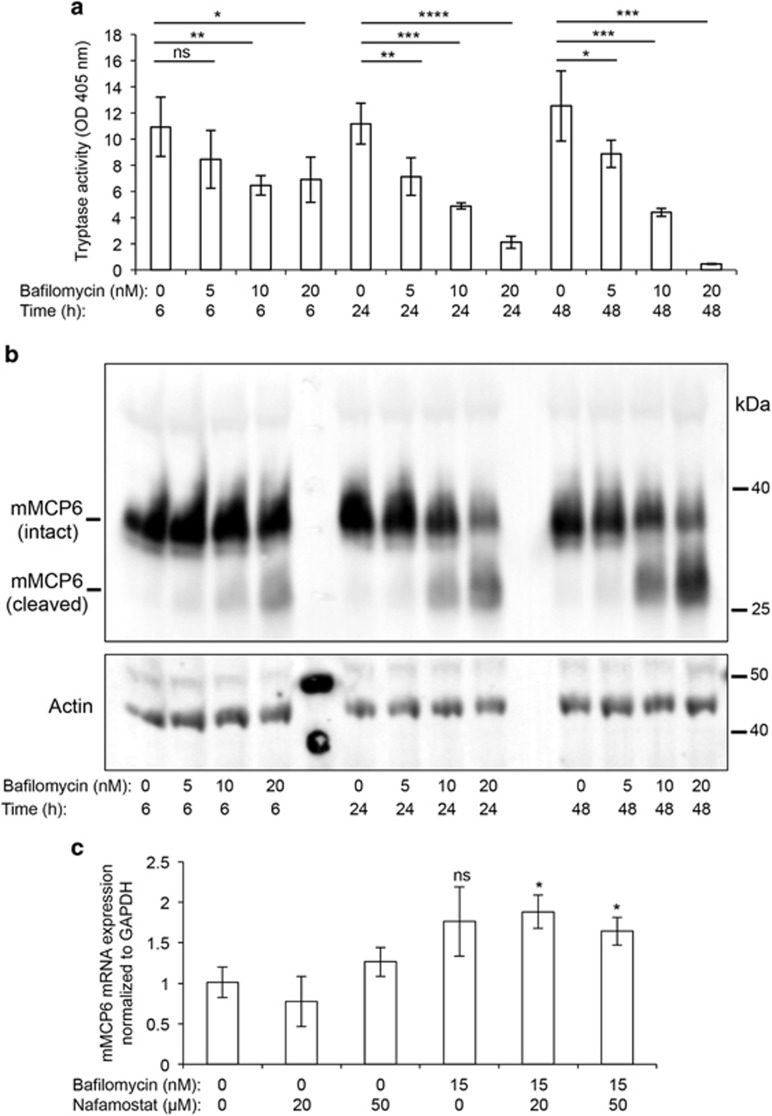
Impaired granule acidification reduces the granule content of enzymatically active tryptase. Mast cells (1 × 10^6^ cells/ml) were incubated with bafilomycin A1 at the concentrations and time periods indicated. (**a**) Cell extracts were prepared and analyzed for tryptase activity using a chromogenic substrate. Results are representative of five individual experiments. Results are given as mean values±S.D. (*n*=3). (**b**) Cell extracts were prepared and subjected to western blot analysis using an anti-tryptase (mMCP6) antibody. *β*-actin was used a loading control. (**c**) RNA from vehicle-treated, bafilomycin A1-treated and nafamostat-treated mast cells (0.5 × 10^6^ cells) was analyzed by qPCR for content of mRNA coding for the tryptase gene (*Mcpt6*). Results are given as mean±S.D. (*n*=3). Results are representative of three individual experiments. *P<0.05, **P<0.01, ***P<0.001, ****P<0.001; ns, not significant

Together with CPA3, tryptase is a major protease stored within the granules of mast cells.^13^ Next, we asked whether tryptase is dependent on acidic pH for proper storage in the mast cell granules. As depicted in Figure 7a, incubation of mast cells with bafilomycin A1 caused a profound reduction of tryptase activity, in a dose- and time-dependent manner. Western blot analysis showed that the reduction in tryptase enzymatic activity was accompanied by formation of tryptase degradation products (Figure 7b). However, it was notable that intact tryptase protein was seen even under conditions where almost complete loss of tryptase enzymatic activity was seen (48 h, 20 nM bafilomycin A1), suggesting that the loss of tryptase activity is only partly explained by protein degradation. Bafilomycin A1 treatment did not reduce the expression of the tryptase gene (*Mcpt6*) (Figure 7c).

**Figure 8:**
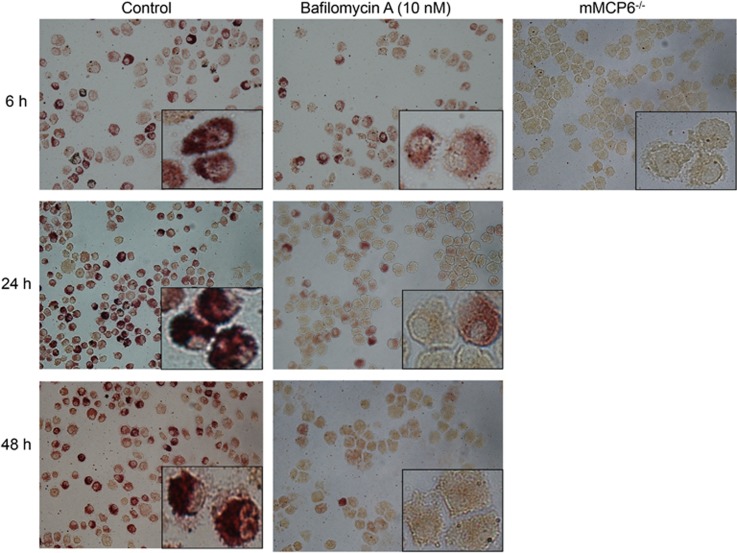
*In situ* detection of tryptase activity after bafilomycin A treatment. Cytospin slides were prepared from cultures of wild-type (**a**) and tryptase-deficient (mMCP6^−/−^) and were stained with fast garnet for detection of trypsin-like activity. Mast cells were either non-treated (control) or incubated with 10 nM bafilomycin A1 for various time periods as indicated, followed by preparation of cytospin slides and fast garnet staining

To further investigate the effects of bafilomycin A1 on tryptase we next implemented a method for *in situ* detection of tryptase activity. For this purpose we used the fast garnet assay, a method that detects trypsin-like activity.^14^ However, the fast garnet method will detect a range of proteases with trypsin-like activity, that is, not just mast cell tryptase, and to evaluate to what extent mast cell tryptase accounts for the total trypsin-like activity in mast cells we therefore subjected both wild-type and tryptase-deficient (mMCP6^−/−^) mast cells to fast garnet staining. As seen in Figure 8, wild-type mast cells stained strongly and the staining showed a distinct granular appearance, in agreement with the location of tryptase within the secretory granules. In contrast, fast garnet staining was undetectable in tryptase-deficient mast cell (Figure 8), showing that the fast garnet technique selectively detects tryptase activity in mast cells. After treatment of wild-type mast cells with bafilomycin A1 (10 nM), a profound decrease in fast garnet staining was seen after 24 h with a further decrease after 48h, whereas only a slight decrease in staining was seen after 6 h (Figure 8). More pronounced effects were seen when increasing the bafilomycin A1 concentration to 20 nM (data not shown). Hence, these data are in strong support of a role for acidic pH in maintaining tryptase activity in the secretory granules of mast cells.

**Figure 9:**
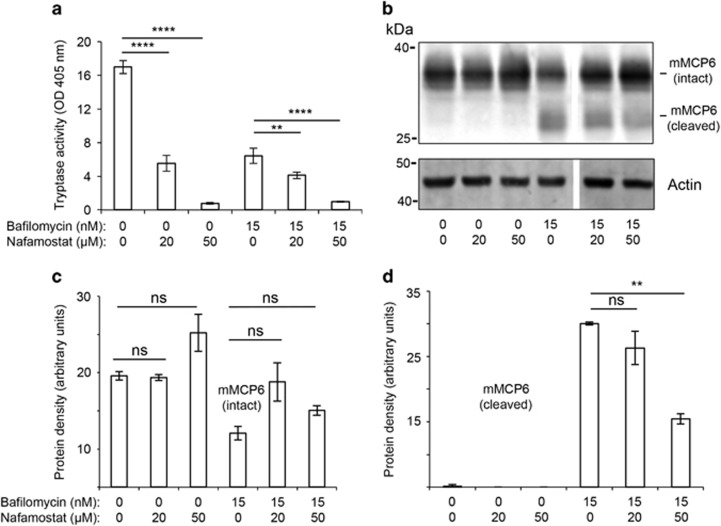
Impaired granule acidification causes autoproteolysis of tryptase. Mast cells (1 × 10^6^ cell/ml) were incubated with bafilomycin A1, either alone or in combination with Nafamostat mesylate (tryptase inhibitor) at the concentrations and time periods indicated. (**a**) Cell extracts were prepared and analyzed for tryptase activity using a chromogenic substrate. Results are representative of three individual experiments. Results are given as mean values±S.D. (*n*=3). (**b**) Cell protein extracts were prepared and subjected to western blot analysis using an anti-tryptase (mMCP6) antibody. *β*-actin was used a loading control. (**c**,**d**) Quantification of the intensities of bands representing intact (**c**) and cleaved (**d**) mMCP6. The results are representative of three individual experiments. Results are given as mean±S.D. (*n*=3). **P<0.01, ****P<0.001; ns, not significant

To search for the mechanism of tryptase degradation we considered the possibility of autoproteolyis. To test this we incubated mast cells with a selective tryptase inhibitor (Nafamostat mesylate).^15^ As expected, incubation of mast cells with Nafamostat mesylate resulted in essentially complete abrogation of tryptase enzymatic activity, and this was seen in both untreated and bafilomycin A1-treated mast cells (Figure 9a). Moreover, it was observed that the incubation of mast cells with Nafamostat mesylate resulted in a reduction in the levels of degraded tryptase (Figure 9b; quantified in Figure 9d) along with a tendency towards an increase in intact tryptase (Figure 9b; quantified in Figure 9c). Hence, these findings suggest that the elevated granule pH in bafilomycin A1-treated mast cells results in autoproteolysis of tryptase.

## Discussion

Research conducted over several decades has provided important insight into the biological function of mast cells in a variety of pathological contexts.^1, 16^ In many of these, mast cell degranulation is a hallmark event and, based on this notion, extensive efforts have been made to elucidate the mechanisms leading to mast cell degranulation and to study the function of the released granule mediators.^4, 17, 18^ Such efforts have revealed a central position of the mediators stored within the mast cell granules in mediating pro-inflammatory effects in the context of many disease settings,^2^ and there is also considerable knowledge of the mechanisms of mast cell degranulation.^18, 19^ However, although the signaling pathways leading to mast cell degranulation have been well studied, there is relatively little knowledge regarding the mechanisms of mast cell granule biogenesis and of the mechanisms that operate in regulating mast cell granule homeostasis (reviewed in references 2, 20). In this study, we addressed one of the important and hitherto unanswered questions related to this topic, namely the role of acidic pH in mast cell granule homeostasis.

Previous investigations have established that mast cell granules have an acidic pH, with estimations ranging from 5.2 to 6.1^7, 8, 9^ and it has been suggested that the acidic pH of the granules may serve to control the activity of the neutral mast cell proteases.^7^ Moreover, based on findings from other types of granule-containing cells,^21^ it can be speculated that the acidic pH serves to generate a force that drives the accumulation of biogenic amines in the mast cell granules. Here we expanded on this issue by testing the direct effects of perturbing the H^+^ transport into the mast cell granules, and show that the inhibition of this process by bafilomycin A1 causes marked effects on mast cell granule homeostasis. First, we noted that bafilomycin A1-treated mast cells develop a massive enlargement of granules, with the granules adopting a vacuole-like morphology. Importantly, these morphological effects were seen in viable cells, indicating that the observed effects were not due to general toxicity of bafilomycin A1 to mast cells. Moreover, we show that elevation of the pH in mast cell granules results in impaired storage of histamine and profound effects on the granule proteases. Notably though, the impaired granule acidification showed selectivity in terms of effects on individual granule compounds, as shown by the profound effects on histamine, CPA3 and tryptase, whereas the storage of *β*-hexosaminidase was unaffected by elevating the pH.

The mechanisms behind our findings are intriguing. With regard to histamine, the observed storage defect could potentially be explained by the effect of pH elevation on its electrical charge. At acidic pH, histamine carries two positive charges and can therefore interact tightly with the acidic proteoglycans of serglycin type that are present in the granules;^22, 23^ as proof of this notion, ablation of serglycin (or of the sulfation of serglycin-associated glycosaminoglycan chains) causes severe defects in the ability of mast cells to store histamine.^24, 25^ However, when the pH is raised, histamine will loose one of its positive charges and detach from the granule proteoglycans. A likely consequence of this could be that the storage of histamine is hampered. Moreover, our data suggest that histamine, after detachment from its proteoglycan partner, is preferentially released from the cells as indicated by the observed increase in extracellular histamine levels after bafilomycin A1 treatment.

On a different angle, effects on histamine could potentially also explain the marked swelling of granules seen after bafilomycin A1-induced elevation of the pH. When histamine is complex-bound to the granule proteoglycans, the concentration of free histamine in the granule compartment will be reduced. However, when the pH is raised, detachment of histamine from proteoglycans will lead to an increase in the concentration of non-complexed histamine. Potentially, this could increase the osmotic pressure of the granules, leading to influx of water and consequent swelling. Interestingly, similar to this study, swelling of granules was also observed in mast cells lacking sulfated glycosaminoglycans, that is, the binding partner for histamine.^25, 26^ It is possible that the lack of binding partner for histamine can cause an increased osmotic pressure within the granules, thereby leading to influx of water and resulting in phenotypic effects similar to those seen as a consequence of pH elevation.

The effects of bafilomycin A1 on proCPA3 processing most likely lies within the properties of the enzymes responsible for the conversion of proCPA3 to active enzyme. Previous studies have suggested that proCPA3 processing into its active form is mediated by the aspartic acid proteases cathepsin E^27^ and there are also indications that granule-localized cysteine cathepsins contribute to this process.^28^ It is notable that these proteases, that is, both the aspartic acid and cysteine cathepsins, have acidic pH optima. It therefore appears likely that the aberrant proCPA3 processing seen in bafilomycin A1-treated mast cells is due to their inactivation as a consequence of the pH elevation.

With regard to our observed effects on tryptase, the underlying mechanism could be more intricate. Tryptase is unique among the mast cell proteases in that it is organized as a tetramer, with all of the active sites facing a relatively narrow central pore.^29^ When organized as a tetramer, tryptase has a very narrow substrate cleavage profile, being essentially unable to act on intact proteins.^29^ Moreover, it has been shown that the tetramer is stabilized by proteoglycans and that the binding to proteoglycans is mediated primarily by electrostatic interactions with histidine residues present on the surface of the tryptase tetramer.^30^ When the pH is elevated, these histidine residues will loose charge leading to detachment of tryptase from its bound proteoglycans, leading to loss of enzymatic activity. Most likely, this mechanism can explain the reduction in the enzymatic activity of tryptase seen after treatment of mast cells with bafilomycin A1. Notably, it was previously demonstrated that, during the process of tryptase destabilization, active tryptase monomers appear transiently.^31^ Such active monomers have a much broader substrate cleavage profile in comparison with tetrameric tryptase.^31, 32^ Potentially, active tryptase monomers would have the ability to cause autoproteolysis and, in agreement with such a scenario, we indeed noted the formation of tryptase degradation products after elevation of the granule pH. Further, in support for an autoproteolytic mechanism, we found that selective inhibition of tryptase using Nafamostat mesylate caused inhibition of the tryptase degradation.

In summary, our present findings establish that granule acidification is an important mechanism to ensure proper mast cell granule homeostasis.

## Materials and methods

### Reagents

Bafilomycin A1 was obtained from InvivoGen (San Diego, CA, USA). Stock solutions of 100 *μ*M (160 μl DMSO added to a 10 μg bafilomycin A1) bafilomycin A1 were prepared. In control experiments, equal amounts of DMSO (vehicle control) as for the bafilomycin A1-treated cells were added. The pH probe Lysosensor Blue DND-167 was purchased from ThermoFisher Scientific (Waltham, MA, USA). Nafamostat mesylate was obtained from Sigma-Aldrich (Steinheim, Germany).

### Cell Culture

BMMCs were established according to an earlier described protocol.^33^ in brief, bone marrow cells from either wild-type or tryptase-deficient (mMCP6^−/−^)^34^ C57BL/6 mice were incubated at 37 °C and 5% CO_2_ in BMMC complete media: Dulbecco's modified Eagle’s medium (DMEM) containing 10% FBS, 30% WEHI-3B-conditoned medium, 1% PEST, 1% l-glutamine and 10 ng/ml IL-3. The medium was changed twice a week. After 4 weeks of incubation, mature and pure BMMC populations were obtained. The animal experiments were approved by the local ethical committee (Uppsala djurförsöksetiska nämnd, Uppsala, Sweden; no C 31/14).

### Cell viability test

Cell viability was examined by the CellTiter-Blue cell viability assay (Promega-Invitrogen, Madison, WI, USA). Ten *μ*l of cell viability reagent was mixed with 90 *μ*l of cell suspension in 96-well plates, followed by incubation for 1 h at 37 °C (5% CO_2)_. Assays were performed in triplicates or quadruplicates. After incubation, the fluorescence was read using a microplates reader (model: TECAN Infinite M200) at 560 nm for excitation and 590 nm for emission.

### Measurement of granule pH

Mast cells (BMMCs) were incubated with bafilomycin A1 or DMSO (as vehicle control) for 3 h. Subsequently, cells were placed in 24-well plates (0.5 × 10^6^ cells/well) and 1*μ*l lysosensor was added and cells incubated for 1 h at 37 °C. Cells were extensively washed in warm PBS and fluorescence was monitored by flow cytometry using a LSR Fortessa flow cytometer (BD Biosciences, San Jose, CA, USA).

### Western blot

The harvested cells were washed twice with ice cold PBS before protein analysis. Equal amounts of cells were used for western blot analysis as described previously.^33^ Anti-mMCP6 and anti-CPA3 antibodies were used.^33^

### Degranulation experiment

Cells were sensitized with IgE anti-dinitrophenyl (DNP) at 0.1 *μ*g/ml overnight. After washing with PBS, cells were resuspended in BMMC medium and stimulated with DNP-HSA at 0.5 *μ*g/ml for 1 h. Cell pellets and media were separated by centrifugation at 300 g for 10 min.

### Protease activity

For tryptase activity, the chromogenic substrate S-2288 (Chromogenix, Milano, Italy) was used, and M-2245 (*N*-(4-methoxyphenylazoformyl)-Phe-OH) (Bachem, Bubendorf, Switzerland) was used to detect CPA3 activity. One half million cells were lysed with 150 *μ*l lysis buffer (2M NaCl and 0.5% Triton-X 100 in PBS) for half an hour on ice. Ten *μ*l lysate was then mixed with 90 *μ*l autoclaved water, followed by adding 20 *μ*l of the respective substrate (from stock solutions in H_2_O: 10 mM S-2288, 1.8 mM M-2245). The activity was monitored by reading the absorbance at 405 nm over 30 min using a Versa max microplate reader (Molecular Devices, Sunnyvale, CA, USA). Each measurement was performed in triplicates or quadruplicates.

### Quantitative real-time PCR (qPCR)

qPCR was performed as described previously.^35^ The RNA isolation kit was from Macherey-Nagel (Düren, Germany), and the cDNA synthesis kit was from Bio-rad (Solna, Sweden). SYBR GreenER SuperMix from Invitrogen (Carlsbad, CA, USA) was used. Glyceraldehyde 3-phosphate dehydrogenase (GAPDH) was used as housekeeping gene. The relative amount of cDNA was determined in triplicates and calculated according to the 2^−ΔΔCT^ method. The primers used were (in 5'→3' direction): GAPDH forward: 5'-TCAACAGCAACTCCCACTCTT-3'; GAPDH reverse: 5'-ACCCTGTTGCTGTAGCCGTAT-3'; Cpa3 forward: 5'-GAAAGTTGCAAGGATTGCCAC-3'; Cpa3 reverse: 5'-ATGCCCAGGTCATAAACCCAG-3'; mMCP6 forward: 5'-AGAACCAGGGCTGTGCTGTCT-3'; mMCP6 reverse: 5'-AGAGGGAGCCACACAATGCAA-3' Hdc forward: 5'-GGATTCTGGGTCAAGGACAAGT-3'; Hdc reverse: 5'-AATGCATGAAGTCCGTGGCT-3'.

### Staining of mast cells

Cytospin slides were prepared (50 000 cells/slide) and were stained with MGG as described.^33^ TEM analysis was performed as described previously using four million cells per sample.^36^ For tryptase staining, cytospin slides were incubated for 30 min with a solution of 10 mM Z-Gly-Pro-Arg 4-methoxy-2-naphtylamine (Sigma-Aldrich) in 0.5 M Tris-HCl (pH 7.5) and 5 mg/ml Fast Garnet GBC sulfate salt (Sigma-Aldrich). Cytospin slides were mounted Slowfade Gold antifade reagent (ThermoFisher Scientific) and inspected by light microscopy.

### Histamine and *β*-hexosaminidase assays

Histamine was quantified using ELISA (DRG Instruments, Marburg, Germany) according to the instructions provided by the manufacturer. *β*-hexosaminidase activity was measured as previously described.^33^

### Statistical analysis

All original data were analyzed by using the Microsoft Office Excel 2010 software. Statistical comparisons were performed using a two-tailed *t*-test with the assumption of unequal variance. For multiple comparisons one-way ANOVA with a Bonferroni correction was performed. *P*-values <0.05 were considered significant. All data were derived from two-five independent experiments and are presented as the mean±S.D. *P<0.05; **P<0.01; ***P<0.001; ****P<0.0001; ns, not statistically significant.
